# FIB-4 Score as a Predictor of Eligibility for Elastography Exam in Patients with Polycystic Ovary Syndrome

**DOI:** 10.3390/biomedicines13081878

**Published:** 2025-08-01

**Authors:** Maciej Migacz, Dagmara Pluta, Kamil Barański, Anna Kujszczyk, Marta Kochanowicz, Michał Holecki

**Affiliations:** 1 Department of Internal, Autoimmune and Metabolic Diseases, Faculty of Medical Sciences in Katowice, Medical University of Silesia, 40-751 Katowice, Poland; holomed@gmail.com; 2 Department of Gynecological Endocrinology, Faculty of Medical Sciences in Katowice, Medical University of Silesia, 40-751 Katowice, Poland; dpluta@sum.edu.pl; 3 Department of Epidemiology, Faculty of Medical Sciences in Katowice, Medical University of Silesia, 40-751 Katowice, Poland; kbaranski@sum.edu.pl; 4 Department of Radiology and Nuclear Medicine, Faculty of Medical Sciences, Medical University of Silesia, 40-751 Katowice, Poland; anna.kujszczyk@sum.edu.pl; 5 Clinical Department of Obstetrics, Gynecology and Gynecological Oncology in Kędzierzyn-Koźle, Roosvelta Str. 2, 47-200 Kędzierzyn-Koźle, Poland; marthakoch@interia.pl

**Keywords:** shear wave elastography, FIB-4, polycystic ovary syndrome, metabolic dysfunction-associated steatotic liver disease, PCOS phenotypes

## Abstract

**Background/objectives:** Polycystic ovary syndrome (PCOS) and metabolic dysfunction-associated steatotic liver disease (MASLD) are common co-morbidities in women of reproductive age. PCOS is highly heterogeneous and is, therefore, divided into four phenotypes. MASLD leads to numerous systemic complications. Studies to date have shown an association between PCOS and MASLD. This study was designed to compare the FIB-4 score (based on age, alanine aminotransferase, aspartate aminotransferase and platelet count) and the results of shear wave elastography in assessing the risk of developing MASLD by patients with PCOS divided by phenotypes. **Methods:** The study enrolled 242 women age 18–35 years with PCOS diagnosed according to Rotterdam criteria, hospitalized at the Department of Gynaecological Endocrinology of the University Clinical Centre in Katowice. The study subjects were assigned to phenotypes A to D. Clinical and biochemical assessments were performed (including androgens and metabolic parameters), and the FIB-4 index was calculated. Liver fibrosis was evaluated by shear wave elastography. To balance the group sizes of phenotypes, oversampling with replacement was applied (PROC SURVEYSELECT, SAS), increasing the number of observations for phenotypes B, C, and D fivefold. Statistical analyses were performed based on data distribution (Shapiro–Wilk test), using ANOVA or the Kruskal–Wallis test with Dunn’s correction. Statistical significance was set at *p* < 0.05. **Results:** The FIB-4 score was the highest in phenotype B patients (0.50 ± 0.15), and the lowest in phenotypes A and C (0.42 ± 0.14). The highest rate of positive elastography findings was recorded in phenotype A patients (34.7%) and the lowest in phenotype C group (13.5%). Significant differences between the phenotypes were also found in terms of androgen levels, insulin, HOMA-IR, and the lipid profile. Among patients with positive elastography, the highest FIB-4 scores were recorded in phenotype C group (0.44 ± 0.06), but the differences between the phenotypes were not statistically significant. **Conclusions:** The FIB-4 score was the highest in phenotype B patients and differed significantly from phenotypes A, C and D. In the elastography exam, the fibrosis index was statistically significantly higher in phenotype A compared to other phenotypes. No correlation was detected between the FIB-4 index and positive elastography. The findings suggest that the FIB-4 index may be used for MASLD screening, but its usefulness as a predictor of eligibility for elastography requires more research.

## 1. Introduction

Polycystic ovarian syndrome (PCOS) is the most common endocrine pathology in females of reproductive age worldwide and can affect up to 21% of women [1]. Importantly, the prevalence of PCOS continues to increase. The Rotterdam criteria are currently used to diagnose PCOS [2], but researchers increasingly recognize the diverse presentation of PCOS by dividing it into four phenotypes: phenotype A is characterized by hyperandrogenism, oligo-anovulation, and polycystic ovaries; phenotype B features hyperandrogenism and oligo-anovulation; phenotype C includes hyperandrogenism and polycystic ovaries; and phenotype D is characterized by oligo-/anovulation and polycystic ovaries [3]. Metabolic dysfunction-associated steatotic liver disease (MASLD, formerly non-alcoholic fatty liver disease, NAFLD) is the most common chronic liver condition affecting more than one-third of the world’s adult population [4]. As a systemic disease, it causes complications that are not limited to liver, leading to cardiovascular conditions, chronic kidney disease, type 2 diabetes, and some extrahepatic cancers [5,6,7]. Studies to date have shown an association between PCOS and MASLD [8,9,10]. The prevalence of MASLD lies between 34 and 70% in patients with coexisting PCOS diagnosis, which is highly elevated compared with the general population (14–34%), even after adjusting for BMI [11]. Factors responsible for liver disorders in patients with PCOS include obesity, hyperinsulinemia, insulin resistance, and hyperandrogenism [12,13]. Diagnostic methods are, therefore, being explored to serve as liver screening tools for MASLD in patients with PCOS. Non-invasive indices for assessing liver damage in PCOS include the Fibrosis-4 (FIB-4) index [based on age, alanine aminotransferase (ALT), aspartate aminotransferase (AST), and platelet count], the BAAT index [calculated from age, body mass index (BMI), ALT, and triglyceride levels], the homeostasis model assessment of insulin resistance (HOMA-IR), and androgen levels [14,15,16,17]. Patients with PCOS have been demonstrated to score higher on all of these indices [18,19,20]. Additionally, recent study has shown that the concentration of anti-müllerian hormone (AMH) may be positively correlated with the concentration of androgens in PCOS [21]. FIB-4 is a simple marker that can act as a “red flag”, helping identify patients at risk of advanced liver fibrosis early and refer them for specialist diagnostics or treatment [22]. The FIB-4 index has high diagnostic accuracy in detecting advanced fibrosis in MASLD and is recommended as the first-line screening step according to AASLD and EASL-EASD-EASO guidelines [23,24]. Other tools that may prove useful as early predictors of MASLD are imaging techniques, in particular shear wave elastography [25]. Elastography is designed for assessing liver stiffness as a surrogate quantitative biomarker of fibrosis burden in chronic liver diseases [26]. In adult patients with MASLD, the assessment of hepatic fibrosis should rely on non-invasive scoring systems derived from combinations of biochemical markers or from combinations of biochemical markers with imaging modalities that evaluate hepatic stiffness and/or fat content. These approaches demonstrate superior diagnostic accuracy compared to conventional liver enzyme measurements, specifically alanine aminotransferase (ALT) and aspartate aminotransferase (AST). This recommendation is based on moderate-quality evidence (level of evidence 2) and is supported by a strong consensus among experts. A sequential, multi-step diagnostic algorithm is recommended. As an initial step, a validated, non-proprietary blood-based fibrosis score—such as FIB-4 index—should be applied. In cases where fibrosis remains suspected or when patients are classified as high risk, a second-line evaluation using established imaging techniques, such as liver elastography, is advised to further delineate the stage of fibrosis. This structured approach carries a strong recommendation, underpinned by level 2 evidence and strong expert agreement [24]. Studies have demonstrated a significant correlation between the FIB-4 index and elastography findings in the detection of liver fibrosis [27]. In studies to date, liver stiffness was demonstrated to be significantly higher in patients with PCOS [28]. Therefore, investigating the relationship between FIB-4 and elastographic findings in PCOS may offer a practical, cost-effective approach to early identification of patients at increased risk of hepatic fibrosis, enabling timely referral and targeted intervention. Different phenotypes of polycystic ovary syndrome (PCOS) show significant differences in metabolic and hormonal parameters and the degree of liver fibrosis, which may influence the individual risk of developing metabolic and liver complications in patients with PCOS. In this study, the FIB-4 score was compared with the results of a shear wave elastography exam in patients diagnosed with PCOS (divided by phenotype) to determine whether the FIB-4 score could be a useful predictor of eligibility for elastography in patients with PCOS. The FIB-4 index was selected from among non-invasive indicators of liver fibrosis based on the results of a previous study, which demonstrated that FIB-4 values were significantly higher in patients with PCOS phenotype B compared to those with phenotype A (with values in phenotype B being similar to those observed in phenotypes C and D). In contrast, in the same study, analysis of the BAAT score did not reveal any statistically significant differences between the individual PCOS phenotypes [29]. Moreover, we compared BMI, androgens, and metabolic parameters (including HOMA-IR) among patients across different PCOS phenotypes to assess their usefulness in predicting MASLD. Additionally, we examined the above parameters in patients with positive elastography results, divided by phenotype, to determine whether any of these parameters correlate with the elastography findings.

## 2. Materials and Methods

The study enrolled 242 patients, age 18–35 years, hospitalized at the Department of Gynaecological Endocrinology of the K. Gibinski University Clinical Centre of the Silesian Medical University in Katowice. The subjects were diagnosed with PCOS according to the Rotterdam consensus and then categorized into phenotypes according to the recognized classification: phenotype A—141 patients, phenotype B—31 patients, phenotype C—40 patients, phenotype D—30 patients. PCOS was diagnosed following a medical history review, gynecological examination, determination of ovarian and adrenal androgen levels, and an ultrasound scan of reproductive organs using Voluson 730 Expert ultrasound system. An interview and physical examination were performed in all study subjects in the morning after 12 h of fasting, including measurements of body weight and height, and then the BMI was calculated according to the WHO criteria. CBC, along with ALT and AST assays, was performed using Cobas Pro analytical unit (Roche, Switzerland). Colorimetry [AU 680 analyzer with Beckman Coulter reagents (Brea, CA, USA)] was used for lipid profile and glucose analysis. The FIB-4 index was estimated as follows: FIB-4 = (Age (years) × AST (IU/L))/(Platelet count (10^9/L) × √ALT (IU/L)). Once these assay results were available, the FIB-4 score was calculated using the eMPendium application. Total and free testosterone, 17-hydroxyprogesterone (17-OH-P), androstenedione, dehydroepiandrosterone (DHEAS), sex hormone binding globulin (SHBG), AMH, and insulin levels were determined immunochemically, using microparticles, a chemiluminescent marker (CMIA), and Abbott reagents (Architect i2000SR; Chicago, IL, USA). Insulin resistance was assessed indirectly using HOMA-IR ratio [fasting serum insulin (uIU/mL) × fasting serum glucose (mmol/L)/22.5]. Liver fibrosis (the fibrosis index) was evaluated in a liver ultrasound scan using shear wave elastography with Hologic Supersonic Mach 30 ultrasound system and a Convex C6-1X type head. Cut-off value for diagnosing liver fibrosis was ≥5.1 kPa.

### Statistical Analysis

Random sampling method with replacement (oversampling with replacement) was used to balance the disproportion in the number of patients with phenotypes B, C, and D relative to phenotype A. The sampling was performed using the PROC SURVEYSELECT procedure in SAS with samprate = 5 to increase fivefold the number of observations in these phenotype groups. As a result, the sample sizes between groups were balanced, and the statistical power of comparative analyses was increased while preserving the value distribution in the original data.

Variable values are presented as means with standard deviation. The Shapiro–Wilk test was used for assessing the distribution of variables. For normal distribution of variables in the compared groups, the parametric ANOVA test was used to assess the differences while preserving the homogeneity of variance validated using Levene’s test. In case of abnormal distribution of variables or heterogeneity of variances in the analyzed groups, the differences between the study groups were analyzed using the Kruskal–Wallis one-way ANOVA non-parametric method test with Dunn’s correction for multiple comparisons. Statistical significance was estimated at *p* < 0.05. Given the oversampling and the non-normal distribution of our data, we mostly chose the Kruskal–Wallis test, which is more robust in detecting differences in distributions rather than focusing solely on differences in mean values. This approach helps reduce potential bias and the risk of incorrectly rejecting the null hypothesis when there is no real difference. The description of specific test values is added as Supplementary Information (Supplementary Material Tables S1–S3). The statistical analysis was performed using Statistica 12.0 PL (StatSoft Polska, Kraków, Poland) and SAS 9.3 software (Institute Inc. Cary, NC, USA).

## 3. Results

The study analyzed selected indices and parameters of patients with PCOS representing various phenotypes.

The highest BMI was recorded in phenotype B patients (26.90 ± 7.79), while the lowest BMI was found in in phenotype C patients (24.59 ± 4.69); these differences proved statistically insignificant.

However, significant differences were observed in all of the other parameters analyzed.

As for androgen levels, the highest 17-OH-P concentrations were recorded in phenotype C patients (1.55 ± 0.79), and the lowest in phenotype A patients (1.02 ± 0.45). Statistically significant differences were obtained between phenotypes A vs. B, phenotypes A vs. C, and phenotypes C vs. D. Phenotype A patients (2.24 ± 1.64) and phenotype D patients (1.07 ± 0.66) had the highest and the lowest free testosterone levels, respectively; statistically significant differences were detected between phenotypes A, B, C vs. D. Similarly, total testosterone also varied statistically significantly. The highest androstenedione levels were found in patients with phenotype C (2.45 ± 1.62) and the lowest in patients with phenotype D (1.60 ± 0.75). Statistically significant differences were obtained between phenotypes A vs. B, phenotypes A vs. D, phenotypes B vs. C, and phenotypes C vs. D. The highest DHEAS levels were found in phenotype C patients (364.36 ± 163.11), and the lowest in phenotype D patients (227.71 ± 72.34). Statistically significant differences were detected between phenotypes A, B, C vs. D.

The highest AMH levels were detected in phenotype A patients (7.56 ± 6.50) and the lowest in phenotype B patients (4.07 ± 3.11). Statistically significant differences were detected between phenotypes A vs. B, phenotypes A vs. C, phenotypes B vs. C, phenotypes B vs. D, and phenotypes C vs. D.

As for metabolic parameters, the highest fasting insulin levels were detected in phenotype B patients (10.56 ± 7.69) and the lowest in phenotype C patients (7.08 ± 4.78). Statistically significant differences were revealed between phenotypes A vs. C, phenotypes B vs. C, and phenotypes B vs. D. The highest fasting glucose was found in phenotype B patients (91.20 ± 12.97) and the lowest in phenotype C patients (85.41 ± 6.32). Statistically significant differences were revealed between phenotypes A, C, D vs. B. In terms of HOMA-IR, the highest score was documented in phenotype B patients (2.45 ± 2.17), and the lowest in phenotype C patients (1.52 ± 1.06). Similarly to insulin, significant differences were found between phenotypes A vs. C, phenotypes B vs. C, and phenotypes B vs. D. The highest total cholesterol levels were detected in phenotype D patients (181.91 ± 33.20), and the lowest in phenotype C patients (162.82 ± 28.22). Statistically significant differences were identified between phenotypes A vs. C, phenotypes B vs. C, phenotypes B vs. D, and phenotypes C vs. D. The highest LDL levels were found in phenotype D patients (102.06 ± 25.71), and the lowest in phenotype C patients (90.94 ± 26.10). Statistically significant differences were identified between phenotypes A, B, D vs. C. The lowest HDL levels were found in phenotype B patients (53.05 ± 14.05), and the highest in phenotype D patients (61.14 ± 13.74). Statistically significant differences were identified in phenotypes A vs. B, phenotypes B vs. D, and phenotypes C vs. D. The highest triglyceride levels were detected in phenotype B patients (97.87 ± 43.21), and the lowest in phenotype C patients (81.56 ± 23.09); significant differences were identified only between these two groups.

The highest FIB-4 score was obtained in phenotype B patients (0.50 ± 0.15), and the lowest in phenotypes A and C (0.42 ± 0.14). Statistically significant differences were identified between phenotypes B vs. A, C, and D. A positive Fibrosis index was most prevalent in phenotype A patients (49; 34.7%), and the lowest in phenotype C patients (19; 13.5%). Significant differences in the prevalence of fibrosis index were detected between phenotypes A vs. B, C, and D.

Mean values along with standard deviations of selected indices and parameters are presented in Table 1; Table 2 presents statistical significance levels.

Selected indices and parameters were additionally analyzed in subgroups of patients with an all-positive fibrosis index.

In patients with a positive fibrosis index, the highest BMI values were found in phenotype B group (30.35 ± 10.81), and the lowest in phenotype C group (22.18 ± 1.78). There were no significant differences in BMI between PCOS phenotypes.

As for androgen concentrations, the highest 17-OH-P levels (1.43 ± 1.01) were identified in phenotype D patients, and the lowest in phenotype B patients (0.85 ± 0.46). There were no significant differences in the 17-OH-P levels between the study groups. The highest free testosterone levels were found in patients with phenotype A (2.13 ± 1.28), and the lowest in phenotype D patients (0.95 ± 0.24). Significant differences were found between phenotypes A vs. D and between phenotypes B vs. D. The highest total testosterone levels were found in patients with phenotype A (0.40 ± 0.18), and the lowest in phenotype D patients (0.24 ± 0.15). Significant differences were identified between phenotypes A, B, C vs. D. The highest androstenedione levels were identified in patients with phenotype A (1.79 ± 0.73), and the lowest in phenotype B patients (1.29 ± 0.23). Statistically significant differences were identified between phenotypes A vs. B, and between phenotypes A vs. D. The highest DHEAS levels were detected in patients with phenotype C (398.85 ± 171.88), and the lowest in phenotype D patients (215.70 ± 72.53). Significant differences were detected between phenotypes A, B, C vs. D.

The highest AMH levels were recorded in phenotype D patients (7.61 ± 3.26), and the lowest in phenotype B patients (3.30 ± 2.85). Significant differences were found between phenotypes A, C, D vs. B.

As for metabolic parameters, the highest fasting insulin was detected in phenotype B patients (10.71 ± 3.82) and the lowest in phenotype C patients (5.15 ± 3.62). Significant differences were identified between phenotypes A, C, D vs. B. The highest glucose levels were identified in patients with phenotype A (85.25 ± 6.51), and the lowest in phenotype B patients (79.23 ± 7.71). Statistically significant differences were obtained between phenotypes A vs. B. The highest total cholesterol was detected in phenotype D patients (211.68 ± 29.82), and the lowest in phenotype B patients (144.25 ± 25.58). Statistically significant differences were obtained between phenotypes A vs. B and phenotypes A, B, C vs. D. The highest LDL levels were revealed in phenotype D patients (120.26 ± 28.66), and the lowest in in phenotype B patients (75.87 ± 21.80). Statistically significant differences were identified between phenotypes A vs. B and phenotypes A, B, C vs. D. The highest HDL levels were detected in phenotype D patients (66.06 ± 12.99), and the lowest in phenotype B patients (49.41 ± 9.34). Statistically significant differences were identified between phenotypes A vs. B and between phenotypes B vs. D. The highest triglyceride levels were detected in phenotype D patients (126.96 ± 91.14), and the lowest in in phenotype C patients (61.22 ± 17.55). Statistically significant differences were identified between phenotypes A, B, D vs. C. The highest HOMA-IR scores were identified in phenotype B patients (2.04 ± 0.54), and the lowest in phenotype C patients (1.08 ± 0.82). Significant differences were identified between phenotypes B vs. A, C, D and between phenotypes A vs. C.

The highest FIB-4 score was obtained in phenotype C patients (0.44 ± 0.06), and the lowest in phenotype D patients (0.42 ± 0.14). There were no significant differences in the FIB-4 score between the study groups.

Detailed findings for the selected parameters and indices for subjects with an all-positive fibrosis index are listed in Table 3; Table 4 presents statistical significance levels. Figures showing selected indices and parameters in subjects with a positive fibrosis index (in the elastography exam) according to PCOS phenotype are added as Supplementary Information (Supplementary Material Figures S1–S15).

## 4. Discussion

The global prevalence of MASLD was shown to be much higher than previously estimated and continues to increase [30]. MASLD is the most common underlying cause of liver-related morbidity and mortality [31], and it would be reasonable to actively screen for MASLD in patients at risk. The main risk factors for MASLD include obesity (especially visceral obesity), type 2 diabetes, dyslipidaemia, and PCOS [32]. Many researchers have demonstrated that the odds of developing MASLD are higher in patients with PCOS. This is evidenced in a meta-analysis of 32 studies (with a total population of 145,131 patients diagnosed with PCOS and 50,832,503 control subjects). Not only did it reveal a significant association between PCOS and an increased risk of MASLD, but it also demonstrated an increased risk of MASLD in PCOS in both prospective and retrospective studies [8]. Phenotypes delineated by the Rotterdam criteria exhibit heterogeneity in the severity of reproductive and metabolic manifestations. Phenotypes A and B are classified as classic presentations of polycystic ovary syndrome (PCOS), comprising approximately two-thirds of cases. These phenotypes are frequently associated with elevated body mass index (BMI), although BMI distribution may differ according to ethnic background and population-specific factors. Prominent metabolic abnormalities—such as metabolic syndrome, impaired glucose tolerance, and type 2 diabetes mellitus—are commonly observed in these subtypes. Notably, these metabolic disturbances can manifest independently of BMI but are often exacerbated by increased adiposity. Phenotype C, referred to as ovulatory PCOS, is generally considered a milder variant, characterized by relatively preserved ovulatory function, reduced insulin resistance, and a lower prevalence of metabolic co-morbidities compared with phenotypes A and B. In this subgroup, BMI tends to be within normal limits or only modestly elevated; however, progressive increases in BMI may aggravate both reproductive and metabolic dysfunctions and potentially modify the clinical phenotype. Phenotype D, or normoandrogenic PCOS, is defined by chronic anovulation and the presence of polycystic ovarian morphology (PCOM) on ultrasonography, in the absence of biochemical hyperandrogenemia and clinical signs of androgen excess [33]. Factors contributing to MASLD development in PCOS patients are not only obesity and adipose tissue dysfunction but also, among others, insulin resistance (IR), chronic inflammation, and hyperandrogenemia [34]. Insulin resistance, exacerbated by excessive adipose tissue accumulation and the upregulation of adipocyte differentiation, constitutes a central factor not only in the pathogenesis of MASLD but also in the progression of hepatic morbidity and extrahepatic complications. MASLD and PCOS exhibit a bidirectional relationship mediated by insulin resistance and share numerous common pathophysiological characteristics [35]. The association of PCOS with MASLD was demonstrated in the study of Hong SH et al. based on the measurement of MASLD indices, including liver fat score (LFS), fatty liver index (FLI), and hepatic steatosis index (HSI). Additionally, in this study, authors suggested that hyperandrogenism contributes to the progression and/or development of MASLD in PCOS, as the levels of free testosterone and FAI were independently associated with MASLD in PCOS, indicating that they may be important predictors of MASLD [36]. Another study showed that PCOS is an independent risk factor for steatosis and that, IR and hyperandrogenism, this last especially in nonobese patients, are the key players of liver damage in PCOS [37]. There is a related need to increase awareness about MASLD and develop cost-effective diagnostic methods. Currently, liver biopsy remains a gold standard, yet imperfect, in the diagnosis of liver fibrosis [38]. However, as an invasive procedure, liver biopsy carries a risk of complications such as pain, hemorrhage, hypotension, visceral perforation, pneumothorax, and in rare cases, death [39]. In recent years, many researchers have studied non-invasive indices used to assess the extent of liver damage. The FIB-4 index based on age, platelet count, ALT, and AST levels [40] is one of such non-invasive indices. However, there is a consensus between the European Association for the Study of the Liver (EASL) and the American Association for the Study of Liver Diseases (AASLD) that FIB-4 is not a direct marker of liver fibrosis and should not be relied on as a sole diagnostic tool [41]. According to the AASLD, a secondary assessment should be performed in patients with high or indeterminate FIB-4 score, preferentially transient elastography (TE) for liver stiffness measurement (LSM) [23,41]. Alternative imaging methods, including shear wave elastography (SWE), may be considered if TE is unavailable [42]. Two-dimensional SWE was first introduced in Aixplorer diagnostic system (SuperSonic Imagine, Aix-en-Provence, France) [43]. SWE is a non-invasive method for assessing liver stiffness that reveals signs of liver damage [25]. This has been confirmed by several recent studies in which liver stiffness measurements using 2D-SWE strongly correlated with the staging of liver fibrosis determined based on liver biopsy in patients with MASLD [44]. Also, although SWE is routinely used to assess liver stiffness associated with fibrosis of various etiology, it has not been validated for use in the assessment of steatosis [45].

In terms of non-invasive markers of liver damage, Canivet CM et al. have demonstrated that combining two non-invasive tests, such as FIB-4 and TE, can increase the diagnostic accuracy of MASLD compared to using either of these indices alone [46]. Similarly, the Asia-Pacific guidelines also agree that combining imaging procedures with laboratory testing may yield more accurate results than using either technique alone for MASLD, but they do not specify which non-invasive measures are more accurate [47].

Diagnostic tools should be selected based on considerations of not only their availability and cost but also the characteristics of the target population, including the prevailing co-morbidities such as PCOS [42,48,49]. The American Association of Clinical Endocrinology (AACE) recommends testing serum ALT for MASLD in adult patients with PCOS [42]. Higher FIB-4 and BAAT scores were demonstrated in a study by Polyzos SA et al. on a group of 314 patients with PCOS compared to the control group [18]. In a study of PCOS patients divided according to phenotypes, higher FIB-4 index scores were obtained in the group of patients with phenotype B compared to the group with phenotype A, and the group with phenotype B was similar to the groups with phenotype C and D [29]. In another study with a smaller sample size, the FIB-4 index was not consistent with advanced fibrosis, which may be attributed to the young age of the study subjects [50]. Ishiba H et al. highlighted that the various cut-off points of the FIB-4 index were age-related and increased with age. In the youngest subjects, below 49 years of age, the suggested cut-off points ranged from 1.05 to 1.2 [51]. Consequently, more studies are needed to assess the usefulness of the FIB-4 index as a screening tool for MASLD, especially in younger patients, including those with PCOS. However, according to AACE and EASL recommendations, elastography or some other method should preferably be used in this group of patients to screen for MASLD [41,45]. Interesting findings were reported in a study assessing the prevalence of MASLD in a group of 70 subjects with PCOS and control group, based on correlations with the parameters of TE. Higher LSM and controlled attenuation parameter (CAP) values were obtained in subjects with PCOS compared to the control group, although the difference for LSM was not statistically significant. These results suggest that young women with PCOS may be in the early stages of MASLD, which highlights the opportunities for early intervention and prevention strategies to slow MASLD progression and reduce the risk of complications. The preliminary results of this study suggest that CAP and LSM measurements obtained by elastography may be of use as predictive markers of MASLD in women with PCOS [52]. In another study, patients newly diagnosed with PCOS were found to have increased liver stiffness, which correlated with multiple clinical, hormonal, and metabolic parameters, suggesting that liver stiffness may predict metabolic consequences of PCOS at a very early stage [28]. Despite these findings, elastography and liver stiffness measurements are not routinely recommended as screening tools for MASLD in PCOS patients [53].

This study is yet another effort to assess the usefulness of non-invasive liver damage indices in patients with PCOS classified into phenotypes to explore whether any phenotype is associated with increased risk of MASLD.

## 5. Conclusions

In conclusion, following the adjustment of sample sizes for phenotypes B, C, and D to correspond with that of phenotype A, the highest FIB-4 scores were observed in in phenotype B patients, with significant differences between PCOS phenotypes B vs. A, C, D. Interestingly, similar relationships between the study groups were only observed for fasting glucose. In contrast, elastography revealed a statistically significantly higher fibrosis index in patients with phenotype A compared to other phenotypes. Moreover, among all statistically significant parameters, free testosterone, total testosterone, androstenedione, and glucose were found to be the highest in a subgroup of phenotype A patients with an all-positive fibrosis index. In contrast, in a subgroup of patients with an all-positive fibrosis index, no statistically significant values were found for the FIB-4 index, which may suggests that the FIB-4 score has a questionable value in identifying patients with PCOS eligible for elastography. In consideration of the foregoing, screening for MASLD should preferably be considered in patients with PCOS phenotypes A and B. However, the usefulness of non-invasive diagnostic methods for MASLD in patients with PCOS requires further research. The substantial dataset presented in this study may provide a foundation for the development of an automated risk stratification tool. Machine learning or artificial intelligence–based methodologies have the potential to generate more robust and accurate indices for predicting the risk of liver fibrosis in populations with PCOS.

Limitations of our study include limited age range (18–35 years)—results may not be generalizable to older PCOS patients. It is single-center study—all participants were recruited from one clinical center, which may limit the external validity. Insulin resistance was assessed indirectly using HOMA-IR ratio—this is an indirect method and not the gold standard (e.g., hyperinsulinemic-euglycemic clamp). Lack of lifestyle assessment: factors such as diet, physical activity, and stress were not evaluated, yet they may influence metabolic outcomes. Initial imbalance in phenotype group sizes: although statistical oversampling was applied, it may affect the natural distribution and interpretation of results.

Androgen levels and metabolic parameters vary among PCOS phenotypes, which buttress the need for individualized clinical care combined with personalized diagnostic and therapeutic strategies to reflect the distinctive characteristics of a specific PCOS phenotype. Future research should focus on multi-center studies involving larger and more diverse populations to validate the current findings and improve their generalizability. Long-term follow-up of patients with different PCOS phenotypes is needed to better understand the progression and risk of developing metabolic and cardiovascular complications. More accurate methods for assessing insulin resistance and liver fibrosis, such as the hyperinsulinemic-euglycemic clamp or liver biopsy, should be incorporated in future studies. Additionally, evaluating the role of lifestyle factors—including diet, physical activity, and stress—could provide valuable insights into phenotype-specific disease mechanisms and management strategies. Interventional studies tailored to individual phenotypes, testing the effectiveness of treatments such as metformin, dietary modifications, or supplements, are also warranted. Finally, the identification and use of novel biomarkers, including adipokines, inflammatory cytokines, or microRNAs, may enhance phenotype classification and help predict long-term outcomes in women with PCOS.

## Figures and Tables

**Table 1:** Selected indices and parameters of all study subjects according to PCOS phenotype.

Index/Parameter	Phenotype				*p*-Value
	A	B	C	D	
Body mass index [kg/m^2^] 17-OH-P [ng/mL] Free testosterone [pg/mL]	25.47 ± 5.40	26.90 ± 7.79	24.59 ± 4.69	25.07 ± 5.35	0.3
	1.02 ± 0.45	1.28 ± 0.64	1.55 ± 0.79	1.23 ± 0.70	<0.0001
	2.24 ± 1.64	1.86 ± 1.08	1.81 ± 1.18	1.07 ± 0.66	<0.0001
Total testosterone [ng/mL] Androstenedione [ng/mL]	0.41 ± 0.17	0.35 ± 0.12	0.39 ± 0.17	0.25 ± 0.11	<0.0001
	1.77 ± 0.71	1.86 ± 1.75	2.45 ± 1.62	1.60 ± 0.75	<0.0001
DHEAS [µg/dL] AMH [ng/mL] Insulin 0′ [µU/mL] Glucose 0′ [mg/dL]	323.80 ± 118.47	334.36 ± 93.99	364.36 ± 163.11	227.71 ± 72.34	<0.0001
	7.56 ± 6.50	4.07 ± 3.11	5.31 ± 3.07	6.13 ± 3.53	<0.0001
	8.95 ± 5.16	10.56 ± 7.69	7.08 ± 4.78	8.36 ± 7.54	<0.0001
	85.60 ± 6.04	91.20 ± 12.97	85.41 ± 6.32	86.22 ± 6.35	<0.0001
Total cholesterol [mg/dL] LDL [mg/dL]	178.32 ± 33.69	170.83 ± 29.80	162.82 ± 28.22	181.91 ± 33.20	<0.0001
	100.60 ± 29.23	98.22 ± 23.41	90.94 ± 26.10	102.06 ± 25.71	0.0005
HDL [mg/dL]	58.46 ± 14.77	53.05 ± 14.05	55.70 ± 10.79	61.14 ± 13.74	0.001
Triglyceride [mg/dL]	96.88 ± 48.95	97.87 ± 43.21	81.56 ± 23.09	96.40 ± 58.74	<0.0001
HOMA-IR [-]	1.92 ± 1.15	2.45 ± 2.17	1.52 ± 1.06	1.80 ± 1.66	0.04
FIB-4 [-]	0.42 ± 0.14	0.50 ± 0.15	0.42 ± 0.14	0.44 ± 0.14	<0.0001
Fibrosis Index [-]	49; 34.7%	24; 17.0%	19; 13.5%	28; 19.8%	<0.0001

**Table 2:** Selected indices and parameters of all study subjects according to PCOS phenotype—between-group statistical significance levels.

Index/Parameter	Levels of Statistical Significance Between Phenotypes [*p*]					
	A vs. B	A vs. C	A vs. D	B vs. C	B vs. D	C vs. D
Body mass index [kg/m^2^] 17-OH-P [ng/mL] Free testosterone [pg/mL]	1	1	1	0.5	1	1
	0.001	<0.0001	0.2	0.2	0.7	0.002
	1	0.4	<0.0001	1	<0.0001	<0.0001
Total testosterone [ng/mL] Androstenedione [ng/mL]	0.06	1	<0.0001	1	<0.0001	<0.0001
	0.03	0.3	0.02	<0.0001	1	<0.0001
DHEAS [µg/dL] AMH [ng/mL] Insulin 0′ [µU/mL] Glucose 0′ [mg/dL]	1	0.6	<0.001	1	<0.0001	<0.0001
	<0.0001	0.001	0.5	<0.0001	<0.0001	0.2
	1	0.01	0.1	<0.0001	0.007	1
	0.0005	1	1	<0.001	0.01	1
Total cholesterol [mg/dL] LDL [mg/dL]	0.7	<0.0001	1	0.03	0.04	<0.0001
	1	0.01	1	0.01	1	<0.001
HDL [mg/dL]	0.03	1	0.6	0.7	<0.0001	0.02
Triglyceride [mg/dL]	1	0.4	1	0.06	0.2	1
HOMA-IR [-]	0.9	0.01	0.2	<0.0001	0.002	1
FIB-4 [-]	<0.0001	1	0.7	<0.0001	0.001	1
Fibrosis Index [-]	<0.001	<0.0001	0.005	0.4	0.5	0.1

**Table 3:** Selected indices and parameters in subjects with a positive fibrosis index (in the elastography exam) according to PCOS phenotype.

Index/Parameter	Phenotype				*p*-Value
	A	B	C	D	
Body mass index [kg/m^2^] 17-OH-P [ng/mL] Free testosterone [pg/mL]	24.51 ± 6.24	30.35 ± 10.81	22.18 ± 1.78	24.49 ± 6.16	0.09
	1.06 ± 0.50	0.85 ± 0.46	1.27 ± 0.66	1.43 ± 1.01	0.09
	2.13 ± 1.28	1.65 ± 0.84	1.57 ± 0.63	0.95 ± 0.24	0.0001
Total testosterone [ng/mL] Androstenedione [ng/mL]	0.40 ± 0.18	0.37 ± 0.10	0.39 ± 1.69	0.24 ± 0.15	<0.0001
	1.79 ± 0.73	1.29 ± 0.23	1.69 ± 0.46	1.38 ± 0.53	0.001
DHEAS [µg/dL] AMH [ng/mL] Insulin 0′ [µU/mL] Glucose 0′ [mg/dL]	304.11 ± 111.64	347.96 ± 87.04	398.85 ± 171.88	215.70 ± 72.53	<0.0001
	7.43 ± 4.83	3.30 ± 2.85	5.70 ± 2.14	7.61 ± 3.26	<0.0001
	8.25 ± 5.54	10.71 ± 3.82	5.15 ± 3.62	7.10 ± 3.79	<0.0001
	85.25 ± 6.51	79.23 ± 7.71	82.52 ± 3.96	83.51 ± 4.43	0.04
Total cholesterol [mg/dL] LDL [mg/dL]	172.86 ± 28.59	144.25 ± 25.58	154.79 ± 6.70	211.68 ± 29.82	<0.0001
	95.49 ± 25.95	75.87 ± 21.80	83.88 ± 12.70	120.26 ± 28.66	<0.0001
HDL [mg/dL]	59.37 ± 14.58	49.41 ± 9.34	58.66 ± 9.05	66.06 ± 12.99	<0.0001
Triglyceride [mg/dL]	89.96 ± 50.17	94.85 ± 23.95	61.22 ± 17.55	126.96 ± 91.14	0.0002
HOMA-IR [-]	1.76 ± 1.24	2.04 ± 0.54	1.08 ± 0.82	1.49 ± 0.82	<0.0001
FIB-4 [-]	0.41 ± 0.16	0.42 ± 0.14	0.44 ± 0.06	0.38 ± 0.14	0.4

**Table 4:** Selected indices and parameters in subjects with a positive fibrosis index (in the elastography exam) according to PCOS phenotype—between-group statistical significance levels.

Index/Parameter	Levels of Statistical Significance Between Phenotypes [*p*]					
	A vs. B	A vs. C	A vs. D	B vs. C	B vs. D	C vs. D
Body mass index [kg/m^2^] 17-OH-P [ng/mL] Free testosterone [pg/mL]	0.2	1	1	0.1	0.3	1
	1	1	1	0.1	0.2	1
	1	1	<0.001	1	0.02	0.05
Total testosterone [ng/mL] Androstenedione [ng/mL]	1	1	0.002	1	0.01	0.003
	0.007	1	0.01	0.08	1	0.1
DHEAS [µg/dL] AMH [ng/mL] Insulin 0′ [µU/mL] Glucose 0′ [mg/dL]	0.8	1	0.007	1	0.0002	<0.001
	<0.001	1	1	0.02	<0.0001	0.6
	0.008	0.06	1	<0.0001	0.006	0.3
	0.03	1	1	1	0.4	1
Total cholesterol [mg/dL] LDL [mg/dL]	0.001	0.3	<0.001	1	<0.0001	<0.0001
	0.03	0.8	0.001	1	<0.0001	<0.0001
HDL [mg/dL]	0.009	1	0.1	0.06	<0.0001	0.4
Triglyceride [mg/dL]	0.5	0.03	0.2	0.001	1	<0.001
HOMA-IR [-]	0.03	0.03	1	<0.0001	0.02	0.2
FIB-4 [-]	1	1	1	1	1	0.6

## Data Availability

The datasets generated during and/or analyzed during the current study are available at the Department of Gynecological Endocrinology, Faculty of Medical Sciences in Katowice, Medical University of Silesia in Katowice, Katowice, Poland. In the case of data requests, we kindly ask that Maciej Migacz, MD, be contacted.

## Supplementary Materials

The following supporting information can be downloaded at: https://www.mdpi.com/article/10.3390/biomedicines13081878/s1, Figures S1–S15: Figures showing selected indices and parameters in subjects with a positive fibrosis index (in the elastography exam) according to PCOS phenotype (between-group statistical significance levels below); Tables S1–S3: The description of specific test values.

## References

[1] Deswal R., Narwal V., Dang A., Pundir C.S. (2020). The Prevalence of Polycystic Ovary Syndrome: A Brief Systematic Review. J. Hum. Reprod. Sci..

[2] Christ J.P., Cedars M.I. (2023). Current Guidelines for Diagnosing PCOS. Diagnostics.

[3] Rosenfield R.L., Ehrmann D.A. (2016). The Pathogenesis of Polycystic Ovary Syndrome (PCOS): The Hypothesis of PCOS as Functional Ovarian Hyperandrogenism Revisited. Endocr. Rev..

[4] Miao L., Targher G., Byrne C.D., Cao Y.Y., Zheng M.H. (2024). Current status and future trends of the global burden of MASLD. Trends Endocrinol. Metab..

[5] Targher G., Byrne C.D., Tilg H. (2024). MASLD: A systemic metabolic disorder with cardiovascular and malignant complications. Gut.

[6] Kaya E., Yilmaz Y. (2022). Metabolic-associated Fatty Liver Disease (MAFLD): A Multi-systemic Disease Beyond the Liver. J. Clin. Transl. Hepatol..

[7] Younossi Z.M., Golabi P., Price J.K., Owrangi S., Gundu-Rao N., Satchi R., Paik J.M. (2024). The Global Epidemiology of Nonalcoholic Fatty Liver Disease and Nonalcoholic Steatohepatitis Among Patients with Type 2 Diabetes. Clin. Gastroenterol. Hepatol..

[8] Kui Y., Heng Z., Hongling P. (2023). Association between polycystic ovary syndrome and risk of non-alcoholic fatty liver disease: A meta-analysis. Endokrynol. Pol..

[9] Chen H., Simons P.I.H.G., Brouwers M.C.G.J. (2025). Is cardiovascular disease in PCOS driven by MASLD?. Trends Endocrinol. Metab..

[10] Arvanitakis K., Chatzikalil E., Kalopitas G., Patoulias D., Popovic D.S., Metallidis S., Kotsa K., Germanidis G., Koufakis T. (2024). Metabolic Dysfunction-Associated Steatotic Liver Disease and Polycystic Ovary Syndrome: A Complex Interplay. J. Clin. Med..

[11] Paschou S.A., Polyzos S.A., Anagnostis P., Goulis D.G., Kanaka-Gantenbein C., Lambrinoudaki I., Georgopoulos N.A., Vryonidou A. (2020). Nonalcoholic fatty liver disease in women with polycystic ovary syndrome. Endocrine.

[12] Salva-Pastor N., López-Sánchez G.N., Chávez-Tapia N.C., Audifred-Salomón J.R., Niebla-Cárdenas D., Topete-Estrada R., Pereznuñez-Zamora H., Vidaltamayo-Ramírez R., Báez-Arellano M.E., Uribe M. (2020). Polycystic ovary syndrome with feasible equivalence to overweight as a risk factor for non-alcoholic fatty liver disease development and severity in Mexican population. Ann. Hepatol..

[13] Won Y.B., Seo S.K., Yun B.H., Cho S., Choi Y.S., Lee B.S. (2021). Non-alcoholic fatty liver disease in polycystic ovary syndrome women. Sci. Rep..

[14] McPherson S., Stewart S.F., Henderson E., Burt A.D., Day C.P. (2010). Simple non-invasive fibrosis scoring systems can reliably exclude advanced fibrosis in patients with non-alcoholic fatty liver disease. Gut.

[15] Ratziu V., Giral P., Charlotte F., Bruckert E., Thibault V., Theodorou I., Khalil L., Turpin G., Opolon P., Poynard T. (2000). Liver fibrosis in overweight patients. Gastroenterology.

[16] Salgado A.L., Carvalho Ld Oliveira A.C., Santos V.N., Vieira J.G., Parise E.R. (2010). Insulin resistance index (HOMA-IR) in the differentiation of patients with non-alcoholic fatty liver disease and healthy individuals. Arq. Gastroenterol..

[17] Jabczyk M., Nowak J., Jagielski P., Hudzik B., Kulik-Kupka K., Włodarczyk A., Lar K., Zubelewicz-Szkodzińska B. (2023). Metabolic Deregulations in Patients with Polycystic Ovary Syndrome. Metabolites..

[18] Polyzos S.A., Goulis D.G., Kountouras J., Mintziori G., Chatzis P., Papadakis E., Katsikis I., Panidis D. (2014). Non-alcoholic fatty liver disease in women with polycystic ovary syndrome: Assessment of non-invasive indices predicting hepatic steatosis and fibrosis. Hormones.

[19] Hong X., Guo Z., Yu Q. (2023). Hepatic steatosis in women with polycystic ovary syndrome. BMC Endocr. Disord..

[20] Jones H., Sprung V.S., Pugh C.J., Daousi C., Irwin A., Aziz N., Adams V.L., Thomas E.L., Bell J.D., Kemp G.J. (2012). Polycystic ovary syndrome with hyperandrogenism is characterized by an increased risk of hepatic steatosis compared to nonhyperandrogenic PCOS phenotypes and healthy controls, independent of obesity and insulin resistance. J. Clin. Endocrinol. Metab..

[21] Gałczyńska D., Daniluk J., Buczek-Kutermak A., Pruś P., Pluta D. (2025). Decoding the Relationship Between Polycystic Ovary Syndrome and Hormonal Dependencies of Anti-Müllerian Hormone and Other Markers. Biomedicines.

[22] Blanco-Grau A., Gabriel-Medina P., Rodriguez-Algarra F., Villena Y., Lopez-Martínez R., Augustín S., Pons M., Cruz L.M., Rando-Segura A., Enfedaque B. (2021). Assessing Liver Fibrosis Using the FIB4 Index in the Community Setting. Diagnostics.

[23] Rinella M.E., Neuschwander-Tetri B.A., Siddiqui M.S., Abdelmalek M.F., Caldwell S., Barb D., Kleiner D.E., Loomba R. (2023). AASLD Practice Guidance on the clinical assessment and management of nonalcoholic fatty liver disease. Hepatology.

[24] European Association for the Study of the Liver (EASL), European Association for the Study of Diabetes (EASD), European Association for the Study of Obesity (EASO) (2024). EASL-EASD-EASO Clinical Practice Guidelines on the management of metabolic dysfunction-associated steatotic liver disease (MASLD). J. Hepatol..

[25] Selvaraj E.A., Mózes F.E., Jayaswal A.N.A., Zafarmand M.H., Vali Y., Lee J.A., Levick C.K., Young L.A.J., Palaniyappan N., Liu C.H. (2021). Diagnostic accuracy of elastography and magnetic resonance imaging in patients with NAFLD: A systematic review and meta-analysis. J. Hepatol..

[26] Ozturk A., Olson M.C., Samir A.E., Venkatesh S.K. (2022). Liver fibrosis assessment: MR and US elastography. Abdom. Radiol..

[27] Asif M., Sohaib M., Anwaar W., Ahmed A., Khalid N.T., Tariq H., Jamil M.I. (2024). Correlation Between Transient Elastography and Non-invasive Biomarker Scores for the Detection of Liver Fibrosis. Cureus.

[28] Gulumsek E., Pekoz B.C., Koc A.S., Aslan M.Z., Ali Ozturk H., Arici F.N., Sumbul H.E. (2020). Liver Stiffness Is Increased in Polycystic Ovary Syndrome and Related with Complement C1q/Tumor Necrosis Factor-Related Protein 3 Levels: A Point Shear Wave Elastography Study. Ultrasound Q..

[29] Migacz M., Pluta D., Barański K., Krajewski B., Madej P., Holecki M. (2025). Using non-invasive indicators to screen the PCOS population for liver disease—A single-centre study. Endokrynol. Pol..

[30] Riazi K., Azhari H., Charette J.H., Underwood F.E., King J.A., Afshar E.E., Swain M.G., Congly S.E., Kaplan G.G., Shaheen A.A. (2022). The prevalence and incidence of NAFLD worldwide: A systematic review and meta-analysis. Lancet Gastroenterol. Hepatol..

[31] Chan W.K., Chuah K.H., Rajaram R.B., Lim L.L., Ratnasingam J., Vethakkan S.R. (2023). Metabolic Dysfunction-Associated Steatotic Liver Disease (MASLD): A State-of-the-Art Review. J. Obes. Metab. Syndr..

[32] Michalopoulou E., Thymis J., Lampsas S., Pavlidis G., Katogiannis K., Vlachomitros D., Katsanaki E., Kostelli G., Pililis S., Pliouta L. (2025). The Triad of Risk: Linking MASLD, Cardiovascular Disease and Type 2 Diabetes; From Pathophysiology to Treatment. J. Clin. Med..

[33] Joham A.E., Norman R.J., Stener-Victorin E., Legro R.S., Franks S., Moran L.J., Boyle J. (2022). Teede, H.J. Polycystic ovary syndrome. Lancet Diabetes Endocrinol..

[34] Spremović Rađenović S., Pupovac M., Andjić M., Bila J., Srećković S., Gudović A., Dragaš B., Radunović N. (2022). Prevalence, Risk Factors, and Pathophysiology of Nonalcoholic Fatty Liver Disease (NAFLD) in Women with Polycystic Ovary Syndrome (PCOS). Biomedicines.

[35] Soto A., Spongberg C., Martinino A., Giovinazzo F. (2024). Exploring the Multifaceted Landscape of MASLD: A Comprehensive Synthesis of Recent Studies, from Pathophysiology to Organoids and Beyond. Biomedicines.

[36] Hong S.H., Sung Y.A., Hong Y.S., Song D.K., Jung H., Jeong K., Chung H., Lee H. (2023). Non-alcoholic fatty liver disease is associated with hyperandrogenism in women with polycystic ovary syndrome. Sci. Rep..

[37] Petta S., Ciresi A., Bianco J., Geraci V., Boemi R., Galvano L., Magliozzo F., Merlino G., Craxì A., Giordano C. (2017). Insulin resistance and hyperandrogenism drive steatosis and fibrosis risk in young females with PCOS. PLoS ONE.

[38] Abdelhameed F., Kite C., Lagojda L., Dallaway A., Chatha K.K., Chaggar S.S., Dalamaga M., Kassi E., Kyrou I., Randeva H.S. (2024). Non-invasive Scores and Serum Biomarkers for Fatty Liver in the Era of Metabolic Dysfunction-associated Steatotic Liver Disease (MASLD): A Comprehensive Review from NAFLD to MAFLD and MASLD. Curr. Obes. Rep..

[39] Aljawad M., Yoshida E.M., Uhanova J., Marotta P., Chandok N. (2013). Percutaneous liver biopsy practice patterns among Canadian hepatologists. Can. J. Gastroenterol..

[40] Sterling R.K., Lissen E., Clumeck N., Sola R., Correa M.C., Montaner J., S Sulkowski M., Torriani F.J., Dieterich D.T., Thomas D.L. (2006). Development of a simple noninvasive index to predict significant fibrosis in patients with HIV/HCV coinfection. Hepatology.

[41] Wang J.L., Jiang S.W., Hu A.R., Zhou A.W., Hu T., Li H.S., Fan Y., Lin K. (2024). Non-invasive diagnosis of non-alcoholic fatty liver disease: Current status and future perspective. Heliyon.

[42] Cusi K., Isaacs S., Barb D., Basu R., Caprio S., Garvey W.T., Kashyap S., Mechanick J.I., Mouzaki M., Nadolsky K. (2022). American Association of Clinical Endocrinology Clinical Practice Guideline for the Diagnosis and Management of Nonalcoholic Fatty Liver Disease in Primary Care and Endocrinology Clinical Settings: Co-Sponsored by the American Association for the Study of Liver Diseases (AASLD). Endocr. Pract..

[43] Bercoff J., Tanter M., Fink M. (2020). Supersonic shear imaging: A new technique for soft tissue elasticity mapping. IEEE Trans. Ultrason. Ferroelectr. Freq. Control.

[44] Chimoriya R., Piya M.K., Simmons D., Ahlenstiel G., Ho V. (2020). The Use of Two-Dimensional Shear Wave Elastography in People with Obesity for the Assessment of Liver Fibrosis in Non-Alcoholic Fatty Liver Disease. J. Clin. Med..

[45] Honda Y., Yoneda M., Imajo K., Nakajima A. (2020). Elastography Techniques for the Assessment of Liver Fibrosis in Non-Alcoholic Fatty Liver Disease. Int. J. Mol. Sci..

[46] Canivet C.M., Costentin C., Irvine K.M., Delamarre A., Lannes A., Sturm N., Oberti F., Patel P.J., Decaens T., Irles-Depé M. (2023). Validation of the new 2021 EASL algorithm for the noninvasive diagnosis of advanced fibrosis in NAFLD. Hepatology.

[47] Chitturi S., Wong V.W., Chan W.K., Wong G.L., Wong S.K., Sollano J., Ni Y.H., Liu C.J., Lin Y.C., Lesmana L.A. (2018). The asia-pacific working party on non-alcoholic fatty liver disease guidelines 2017-Part 2: Management and special groups. J. Gastroenterol. Hepatol..

[48] Sarkar M., Terrault N., Chan W., Cedars M.I., Huddleston H.G., Duwaerts C.C., Balitzer D., Gill R.M. (2020). Polycystic ovary syndrome (PCOS) is associated with NASH severity and advanced fibrosis. Liver Int..

[49] Falzarano C., Lofton T., Osei-Ntansah A., Oliver T., Southward T., Stewart S., Andrisse S. (2022). Nonalcoholic Fatty Liver Disease in Women and Girls with Polycystic Ovary Syndrome. J. Clin. Endocrinol. Metab..

[50] Taranto D.O.L., Guimarães T.C.M., Couto C.A., Cândido A.L., Azevedo R.C.S., Mattos F.S., Elias M.L.C., Reis F.M., Rocha A.L.L., Faria L.C. (2020). Nonalcoholic fatty liver disease in women with polycystic ovary syndrome: Associated factors and noninvasive fibrosis staging in a single Brazilian center. Arch. Endocrinol. Metab..

[51] Ishiba H., Sumida Y., Tanaka S., Yoneda M., Hyogo H., Ono M., Fujii H., Eguchi Y., Suzuki Y., Yoneda M. (2018). The novel cutoff points for the FIB4 index categorized by age increase the diagnostic accuracy in NAFLD: A multi-center study. J. Gastroenterol..

[52] Chakraborty S., Ganie M.A., Masoodi I., Jana M., Shalimar Gupta N., Sofi N.Y. (2020). Fibroscan as a non-invasive predictor of hepatic steatosis in women with polycystic ovary syndrome. Indian J. Med. Res..

[53] Teede H.J., Tay C.T., Laven J.J.E., Dokras A., Moran L.J., Piltonen T.T., Costello M.F., Boivin J., Redman L.M., Boyle J.A. (2023). Recommendations from the 2023 international evidence-based guideline for the assessment and management of polycystic ovary syndrome. Eur. J. Endocrinol..

