# Supplementary material for: FIB-4 Score as a Predictor of Eligibility for Elastography Exam in Patients with Polycystic Ovary Syndrome
Source: Biomedicines. 2025 Aug 1;13(8):1878. doi: 10.3390/biomedicines13081878 (PMC12383516; doi:10.3390/biomedicines13081878)
Supplement: Supplementary file 1 [file biomedicines-13-01878-s001.zip › biomedicines-3738944-supplementary.pdf]

Tables S1-S3. The description of specific test values.

Table S1. All.

Variable	Test TKW H(3, N=564)
BMI [kg/m^2] 7	3.415980
17-OH-P [ng/ml] 38	34.076775
Free testosterone [pg/ml] 39	65.88115
Total testosterone [ng/ml] 40	86.53711
Androstenedione [ng/ml] 41	31.93803
DHEAS [μg/dl] 42	99.43650
AMH [ng/ml] 49	68.60350
Insulin 0' [μU/ml] 50	21.52448
Glucose 0' [mg/dl] 54	20.61655
Total cholesterol [mg/dl] 28	33.31347
LDL [mg/dl] 29	17.83002
HDL [mg/dl] 30	21.15543
Triglyceryde [mg/dl] 25	8.091864
HOMA-IR [-] 56	21.15826
FIB-4 [-] 24	33.57412

Table S2. Positive fibrosis index.

Variable	Test TKW H(3, N=120)
BMI [kg/m^2] 7	6.383351
17-OH-P [ng/ml] 38	6.415328
Free testosterone [pg/ml] 39	20.53071
Total testosterone [ng/ml] 40	17.23923
Androstenedione [ng/ml] 41	16.22305
DHEAS [μg/dl] 42	26.81824
AMH [ng/ml] 49	28.85323
Insulin 0' [μU/ml] 50	24.47832
Glucose 0' [mg/dl] 54	7.904825
Total cholesterol [mg/dl] 28	50.98536

LDL [mg/dl] 29	35.57342
HDL [mg/dl] 30	22.31000
Triglyceryde [mg/dl] 25	19.94838
HOMA-IR [-] 56	22.09658
FIB-4 [-] 24	2.763050

Table S3. Negative fibrosis index.

Variable	Test TKW H(3, N=444)
BMI [kg/m^2] 7	2.515263
17-OH-P [ng/ml] 38	36.67664
Free testosterone [pg/ml] 39	47.32231
Total testosterone [ng/ml] 40	67.36166
Androstenedione [ng/ml] 41	22.72479
DHEAS [µg/dl] 42	76.03971
AMH [ng/ml] 49	41.85482
Insulin 0' [µU/ml] 50	12.54867
Glucose 0' [mg/dl] 54	33.47730
Total cholesterol [mg/dl] 28	21.63489
LDL [mg/dl] 29	17.82065
HDL [mg/dl] 30	8.889801
Triglyceryde [mg/dl] 25	8.588326
HOMA-IR [-] 56	14.82304
FIB-4 [-] 24	41.11173

Figures S1-S15. Figures showing selected indices and parameters in subjects with a positive fibrosis index (in the elastography exam) according to PCOS phenotype (between-group statistical significance levels below).

Figure S1.

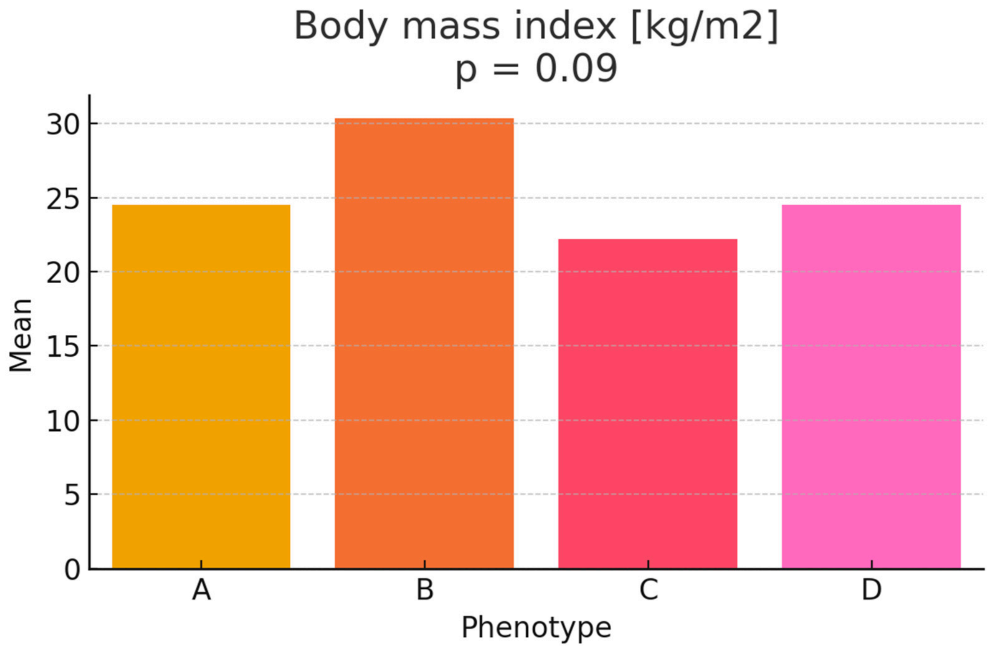

A vs B	A vs C	A vs D	B vs C	B vs D	C vs D
p = 0.2	p = 1	p = 1	p = 0.1	p = 0.3	p = 1

Figure S2.

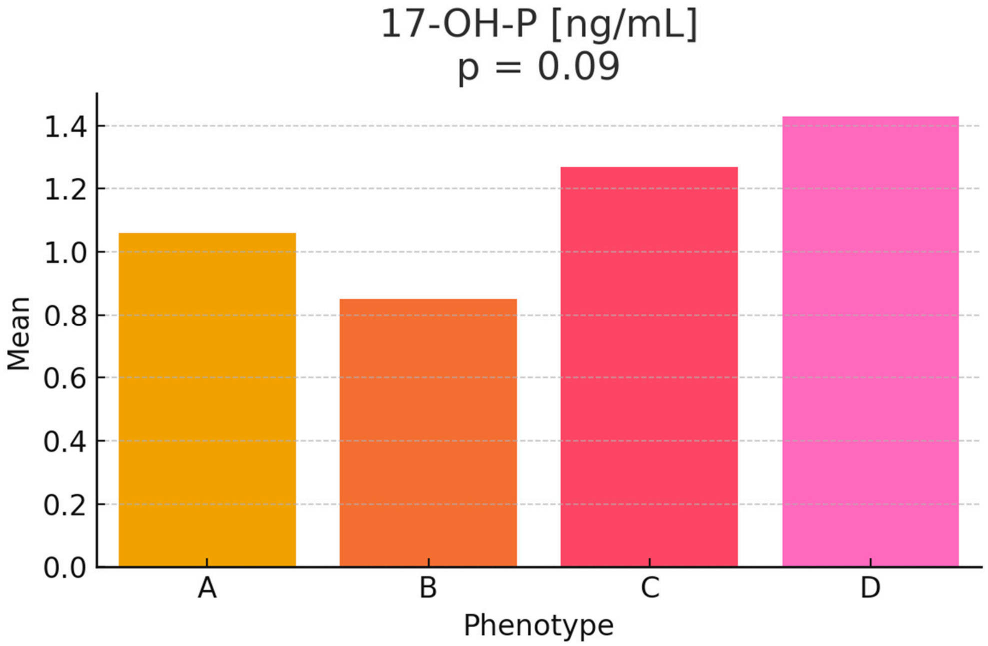

A vs B	A vs C	A vs D	B vs C	B vs D	C vs D
p = 1	p = 1	p = 1	p = 0.1	p = 0.2	p = 1

Figure S3.

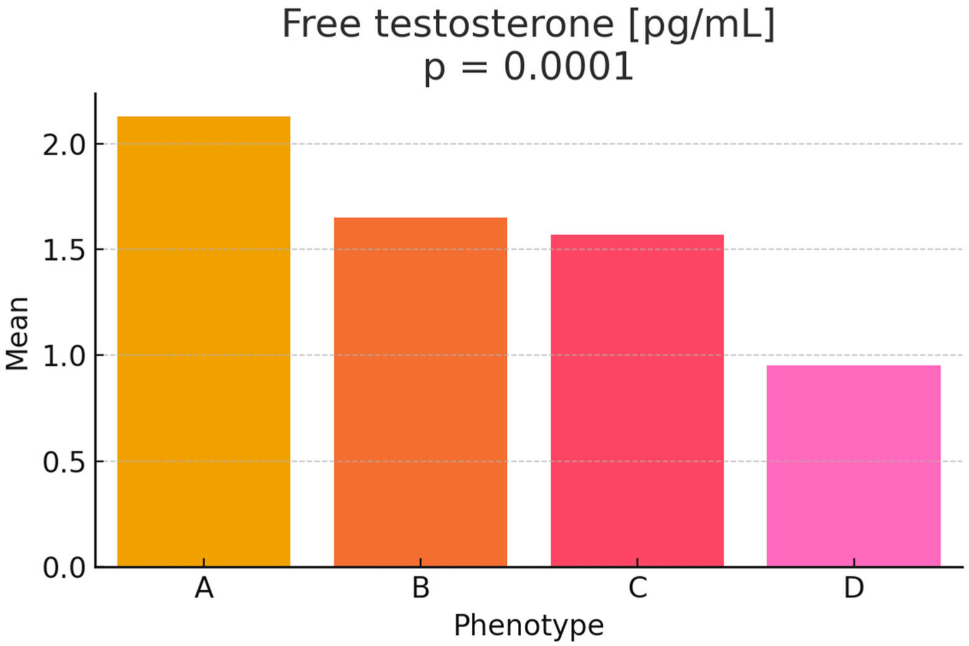

A vs B	A vs C	A vs D	B vs C	B vs D	C vs D
p = 1	p = 1	p = <0.001	p = 1	p = 0.02	p = 0.05

Figure S4.

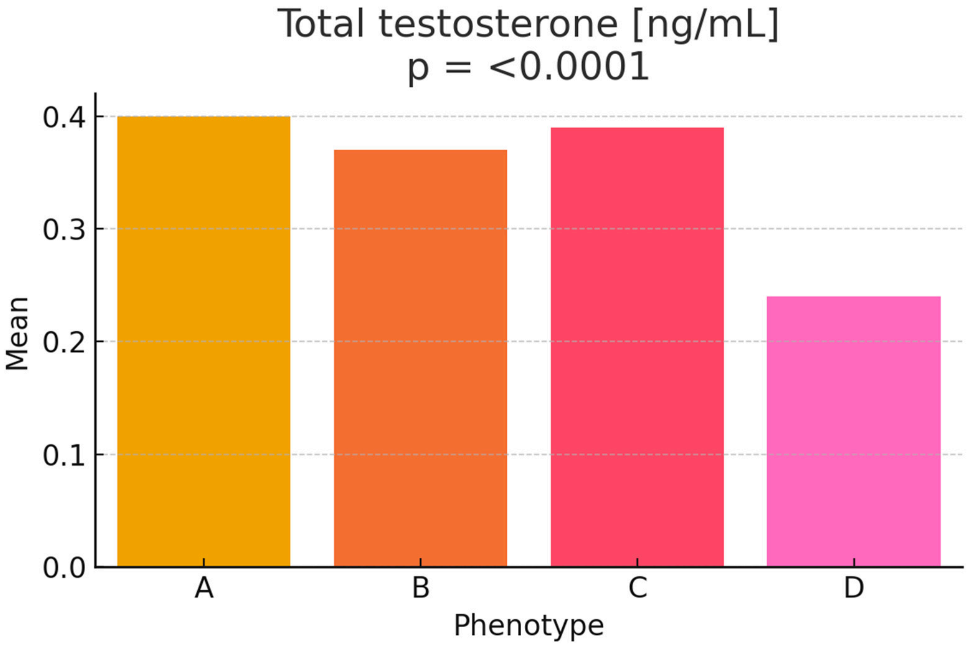

A vs B	A vs C	A vs D	B vs C	B vs D	C vs D
p = 1	p = 1	p = 0.002	p = 1	p = 0.01	p = 0.003

Figure S5.

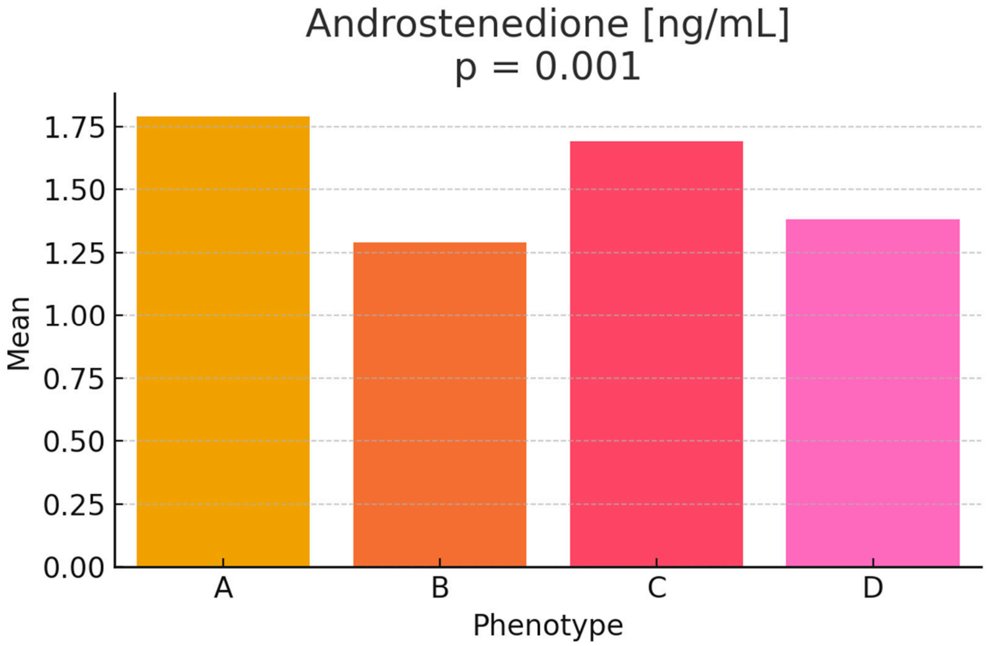

A vs B	A vs C	A vs D	B vs C	B vs D	C vs D
p = 0.007	p = 1	p = 0.01	p = 0.08	p = 1	p = 0.1

Figure S6.

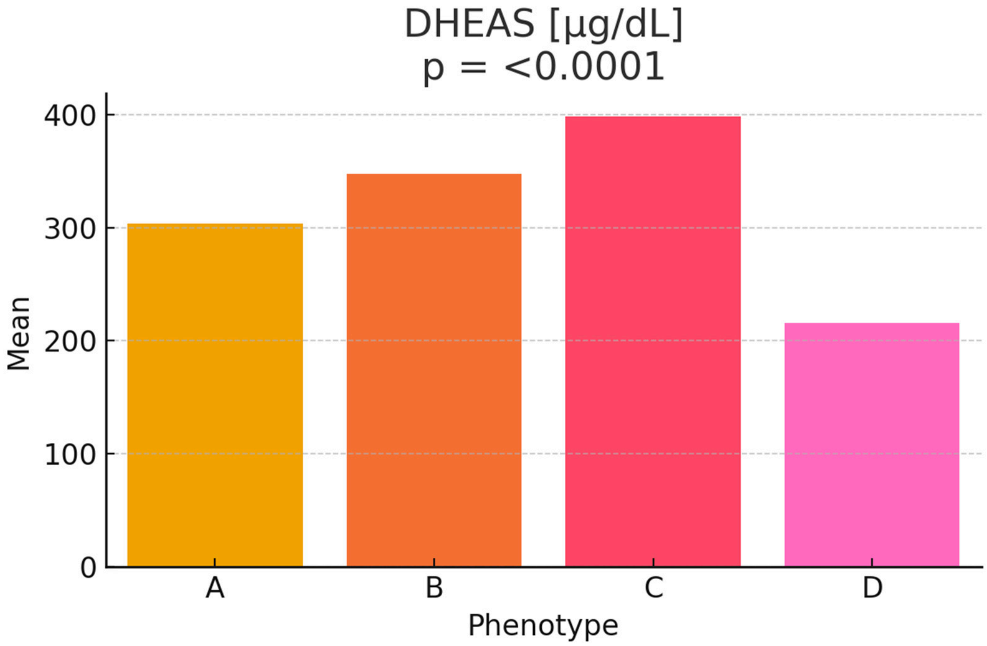

A vs B	A vs C	A vs D	B vs C	B vs D	C vs D
p = 0.8	p = 1	p = 0.007	p = 1	p = 0.0002	p = 0.3

Figure S7.

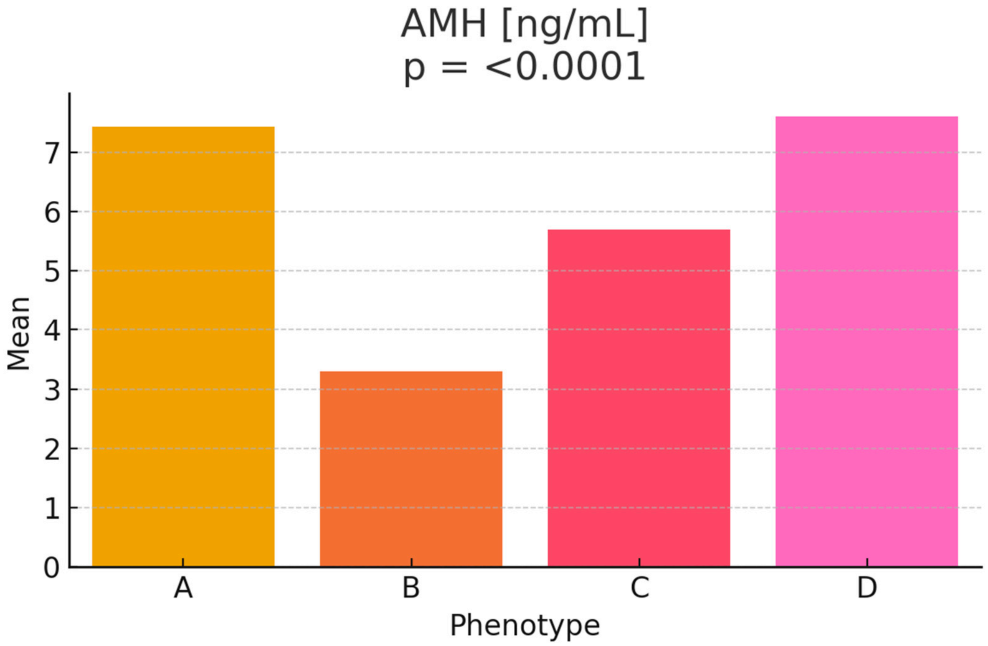

A vs B	A vs C	A vs D	B vs C	B vs D	C vs D
p = <0.001	p = 1	p = 1	p = 0.02	p = <0.0001	p = 0.6

Figure S8.

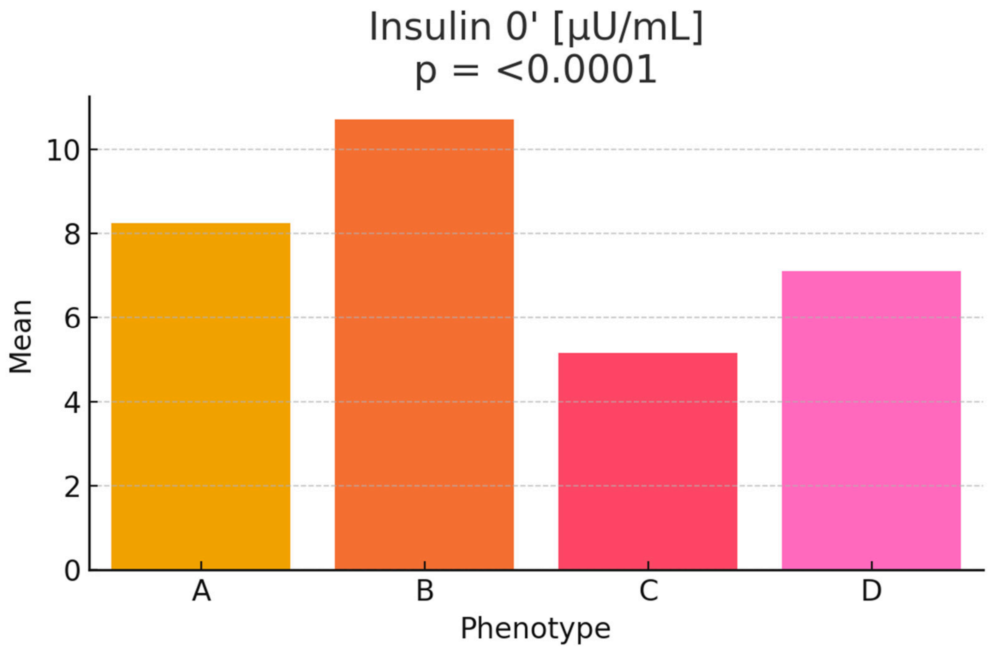

A vs B	A vs C	A vs D	B vs C	B vs D	C vs D
p = 0.008	p = 0.06	p = 1	p = <0.0001	p = 0.006	p = 0.3

Figure S9.

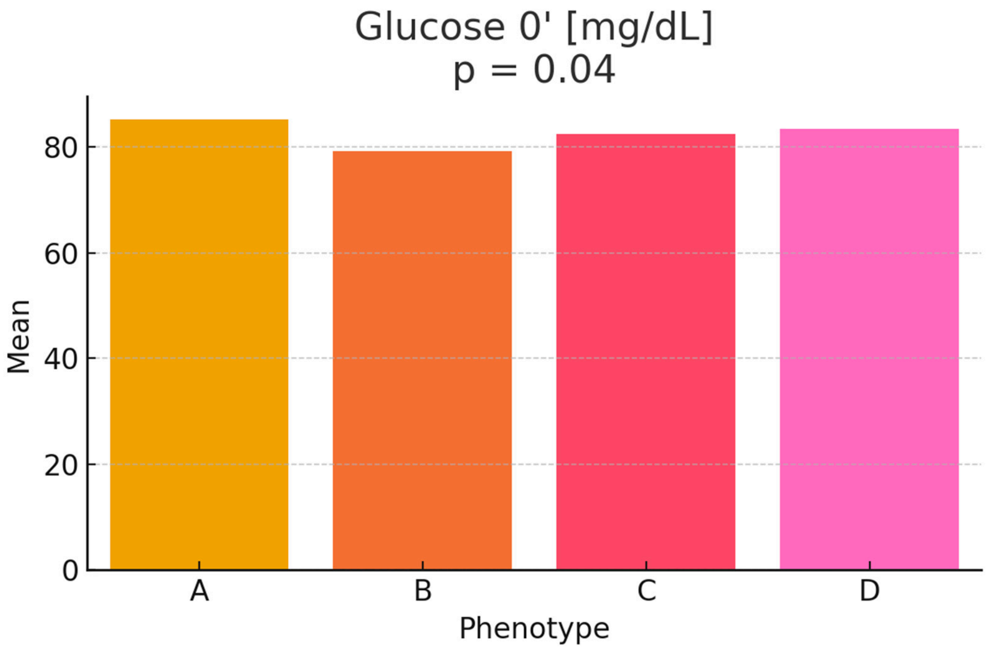

A vs B	A vs C	A vs D	B vs C	B vs D	C vs D
p = 0.03	p = 1	p = 1	p = <0.0001	p = 0.006	p = 1

Figure S10.

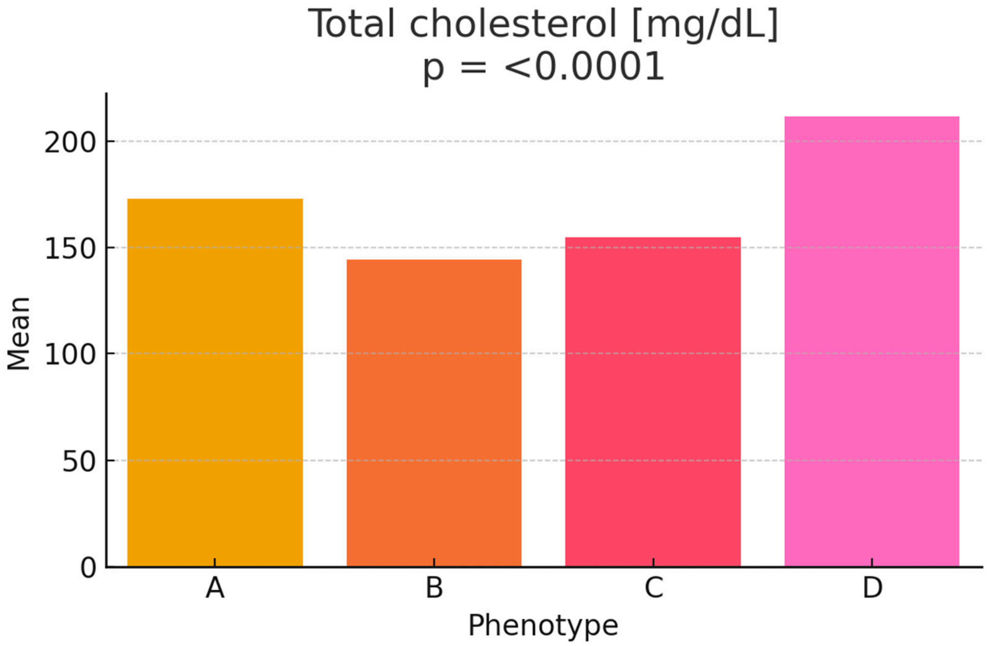

A vs B	A vs C	A vs D	B vs C	B vs D	C vs D
p = 0.001	p = <0.001	p = 0.001	p = 1	p = <0.0001	p = <0.0001

Figure S11.

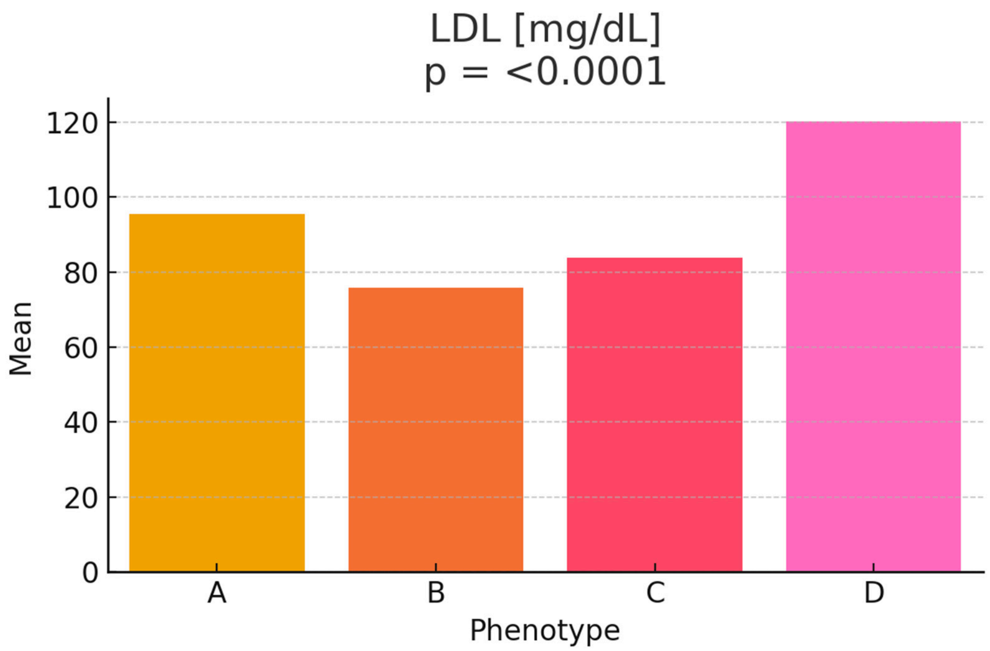

A vs B	A vs C	A vs D	B vs C	B vs D	C vs D
p = 0.03	p = 0.8	p = 0.001	p = 1	p = <0.0001	p = <0.0001

Figure S12.

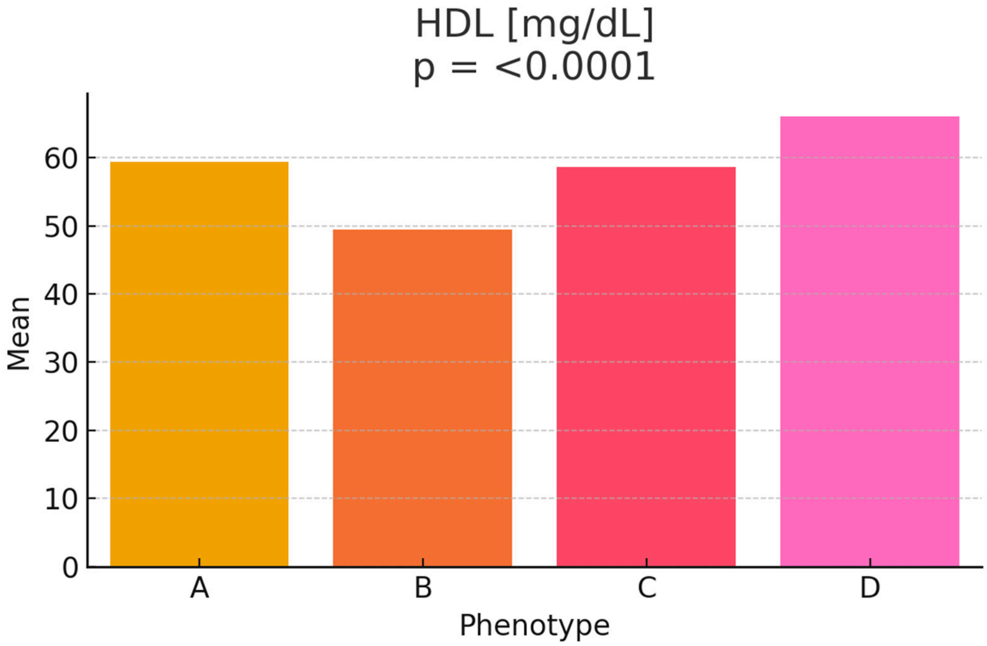

A vs B	A vs C	A vs D	B vs C	B vs D	C vs D
p = 0.009	p = 1	p = 0.1	p = 0.06	p = <0.0001	p = 0.4

Figure S13.

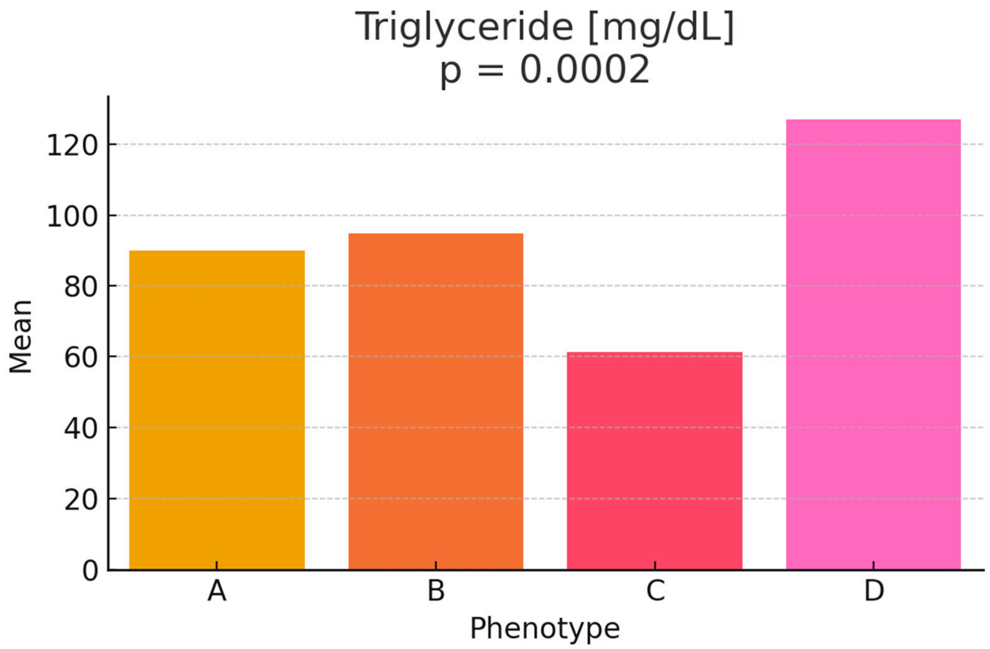

A vs B	A vs C	A vs D	B vs C	B vs D	C vs D
p = 0.5	p = 0.03	p = 0.2	p = 0.001	p = 1	p = <0.001

Figure S14.

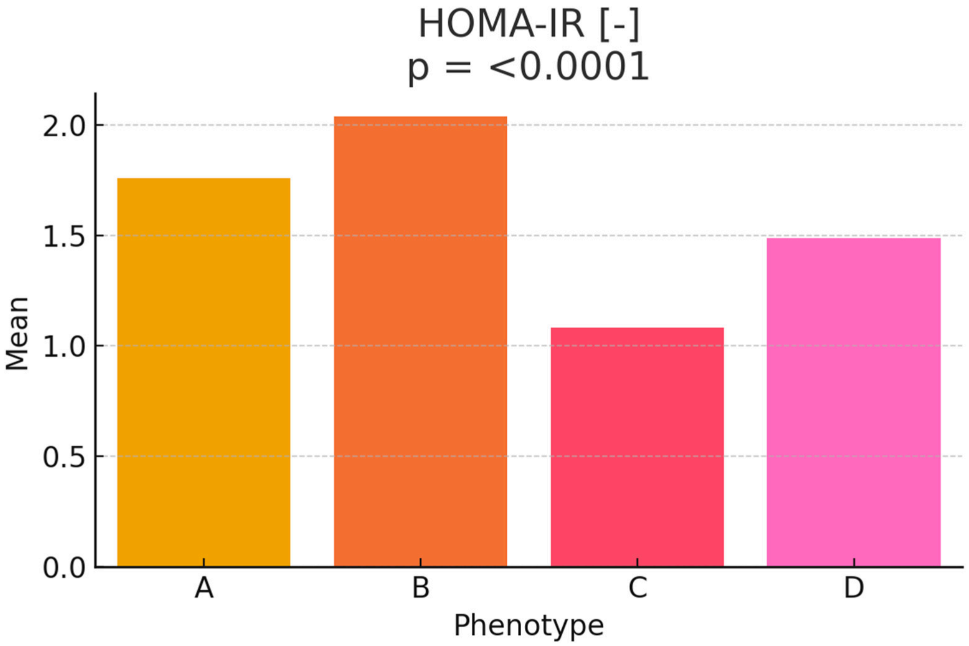

A vs B	A vs C	A vs D	B vs C	B vs D	C vs D
p = 0.03	p = 0.03	p = 1	p = <0.0001	p = 0.02	p = 0.2

Figure S15.

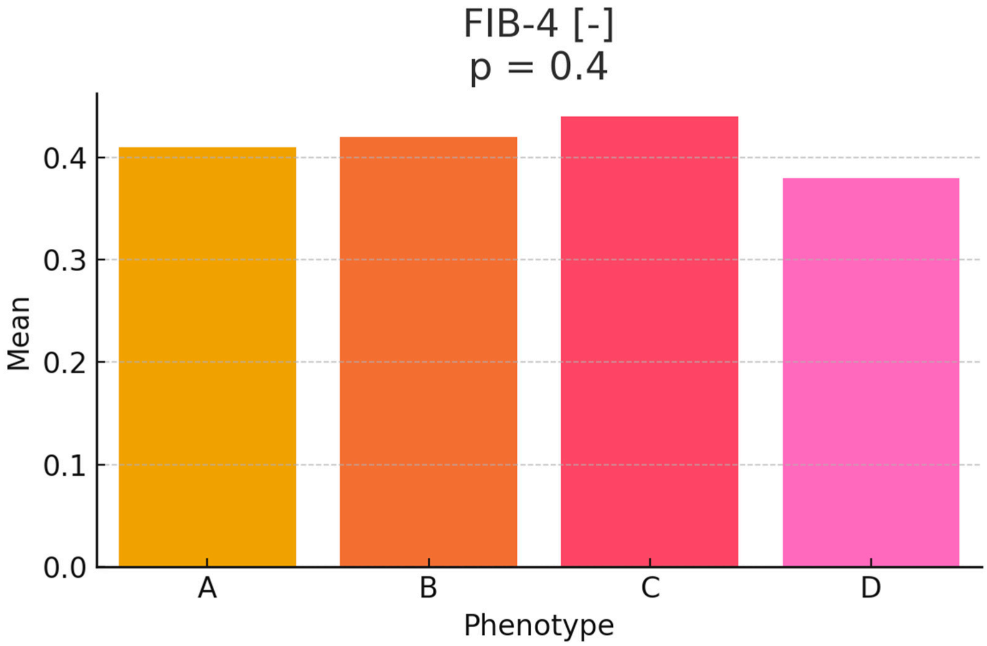

A vs B	A vs C	A vs D	B vs C	B vs D	C vs D
p = 1	p = 1	p = 1	p = 1	p = 1	p = 0.6
